# MRI‐Based Surgical Margin Assessment in Dermatofibrosarcoma Protuberans: Correlation With Pathological Extent

**DOI:** 10.1111/1346-8138.70377

**Published:** 2026-07-03

**Authors:** Munehisa Kito, Mai Iwaya, Toshikazu Omodaka, Shunsuke Yuzuriha, Masanori Okamoto, Atsushi Tanaka, Akira Takazawa, Hirokazu Ideta, Daiki Taruta, Kazuaki Nemoto, Kaoru Aoki, Jun Takahashi

**Affiliations:** ^1^ Department of Orthopaedic Surgery Shinshu University School of Medicine Matsumoto Nagano Japan; ^2^ Department of Laboratory Medicine Shinshu University Hospital Matsumoto Nagano Japan; ^3^ Department of Dermatology Shinshu University School of Medicine Matsumoto Nagano Japan; ^4^ Department of Plastic and Reconstructive Surgery Shinshu University School of Medicine Matsumoto Nagano Japan

**Keywords:** dermatofibrosarcoma protuberans, local recurrence, magnetic resonance imaging, surgical margin, wide local excision

## Abstract

Dermatofibrosarcoma protuberans (DFSP) is a locally aggressive cutaneous tumor characterized by infiltrative growth and a high risk of local recurrence if incompletely excised. Although Mohs micrographic surgery is recommended, wide local excision (WLE) remains widely used in clinical practice; however, the clinical utility of preoperative imaging for surgical margin assessment has not been fully established. We retrospectively analyzed 42 patients with DFSP, including fibrosarcomatous DFSP (FS‐DFSP), who underwent MRI‐based surgical planning followed by WLE between 2009 and 2023. Lateral margins were defined based on preoperative MRI, and deep tumor extent was assessed according to the relationship with the fascia. Analyses were performed in 36 patients after excluding cases without identifiable pathological tumor. The median planned lateral margin was 20 mm, whereas the median pathological margin was 16.5 mm. The median difference (pathological − planned) was −5 mm (interquartile range −10.25 to −1 mm; range −18 to +15 mm), with planned margins significantly larger (*p* < 0.001). A weak positive correlation was observed between planned and pathological margins (*ρ* = 0.32), but it did not reach statistical significance (*p* = 0.060). Importantly, the maximum underestimation of tumor extent was 18 mm, indicating that the discrepancy between imaging‐based planning and pathological findings did not exceed 20 mm in any case. Lesions classified as fascia‐contacting or beyond on MRI showed a higher likelihood of deep invasion (OR 27.0, 95% CI 1.5–483). Residual tumor was identified in 71% of patients undergoing additional resection after unplanned excision. All patients achieved R0 resection without local recurrence, and distant metastases occurred only in FS‐DFSP. Preoperative MRI provides clinically useful information for surgical margin assessment in DFSP. A lateral margin of approximately 20 mm appears sufficient to achieve complete resection, and fascia‐based evaluation may help stratify the risk of deep invasion.

## 1 Introduction

Dermatofibrosarcoma protuberans (DFSP) is a rare, low‐grade cutaneous soft tissue tumor characterized by slow growth but marked local aggressiveness and a high risk of local recurrence if incompletely excised [1, 2, 3, 4]. Although distant metastasis is uncommon, repeated local recurrence may lead to extensive tissue loss and functional or cosmetic impairment [3, 4].

Histopathologically, DFSP exhibits a distinctive infiltrative growth pattern with finger‐like or tentacle‐like extensions that extend beyond the clinically or radiologically apparent tumor boundary [5]. This characteristic makes accurate delineation of tumor margins challenging and has contributed to ongoing debate regarding the optimal surgical margin [6, 7].

Current guidelines, including those from the National Comprehensive Cancer Network (NCCN), recommend Mohs micrographic surgery (MMS) or peripheral and deep en face margin assessment (PDEMA) as the preferred treatment for DFSP [8]. These techniques allow comprehensive evaluation of surgical margins and are associated with low recurrence rates [9, 10, 11]. However, their availability is limited in many clinical settings due to the need for specialized expertise and intraoperative pathological assessment. As a result, wide local excision (WLE) remains widely performed in routine practice [12].

In WLE, the optimal lateral margin remains controversial. While traditional recommendations have suggested wide margins of 2–5 cm [6], more recent evidence indicates that achieving negative margins (R0 resection), rather than the absolute width of the margin, is the key determinant of local control [7, 13]. In contrast, evidence regarding optimal deep surgical margins is limited, and no consensus has been established. From a surgical oncology perspective, anatomical structures such as fascia and periosteum are considered to act as barriers to tumor spread, and margin assessment may be more appropriately based on the relationship between the tumor and these structures rather than on distance alone [14, 15]. However, the clinical utility of preoperative imaging in assessing such relationships and guiding surgical margin planning has not been fully elucidated.

In addition, fibrosarcomatous DFSP (FS‐DFSP) is a histological variant associated with more aggressive biological behavior, including an increased risk of metastasis [12, 16]. Therefore, surgical strategies should also take into account such heterogeneity.

In this study, we retrospectively analyzed patients with DFSP, including FS‐DFSP, to evaluate the relationship between preoperative MRI‐based surgical margin planning and pathological tumor extent. Our aim was to clarify the clinical utility of MRI in surgical margin assessment and to provide practical evidence for optimizing WLE in settings where margin‐controlled techniques are not readily available.

## 2 Patients and Methods

### 2.1 Study Design and Patients

This was a single‐institution retrospective observational study approved by the institutional review board. The requirement for informed consent was waived due to the retrospective nature of the study, and an opt‐out approach was adopted in accordance with the approval of the institutional review board.

A total of 54 patients who underwent surgical resection for DFSP with preoperative MRI‐based surgical planning between 2009 and 2023 were retrospectively identified. Patients with a follow‐up period of < 1 year were excluded, and 42 patients were ultimately included in the analysis.

**FIGURE 1:**
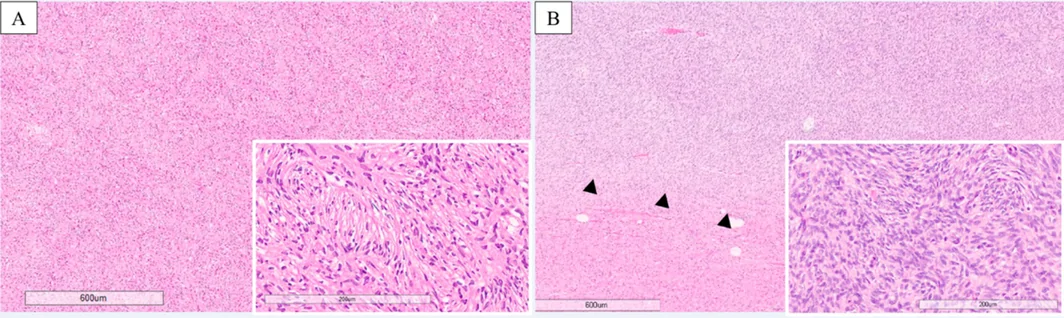
Histology of “typical” dermatofibrosarcoma protuberans (DFSP) (A): Bland spindle cells are distributed in a haphazard, interwoven pattern, relatively uniformly throughout the lesion (inset: high‐power view). DFSP and fibrosarcoma boundary (B): Arrowheads indicate the transition zone between DFSP and fibrosarcoma (inset: high‐power view of the fibrosarcoma component).

The study cohort included both conventional DFSP and fibrosarcomatous DFSP (FS‐DFSP) (Figure 1).

Patients were classified according to their initial clinical presentation into those who underwent primary treatment at our institution and those referred after unplanned excision performed at another institution. Unplanned excision was defined as prior excision without imaging‐based surgical planning. The availability of preoperative imaging before previous excision was also reviewed.

### 2.2 Preoperative Imaging Evaluation and Margin Planning

Lateral surgical margins were determined based on preoperative MRI, primarily using contrast‐enhanced T1‐weighted fat‐suppressed sequences. In cases where contrast‐enhanced sequences were not available, STIR images were used. The tumor boundary was identified on all relevant imaging slices, and the distance from the tumor margin to the planned resection line was measured to define the lateral surgical margin. Tumor size was measured when assessable on preoperative imaging. In cases referred after unplanned excision where no clear tumor lesion was identifiable on imaging, tumor size was recorded as 0 cm but excluded from summary statistics. In cases where the tumor could not be clearly identified on imaging, the resection margin was determined based on areas suggestive of scar tissue or reactive changes, with an additional safety margin. The decision regarding margin width was made at the discretion of the operating surgeon.

**FIGURE 2:**
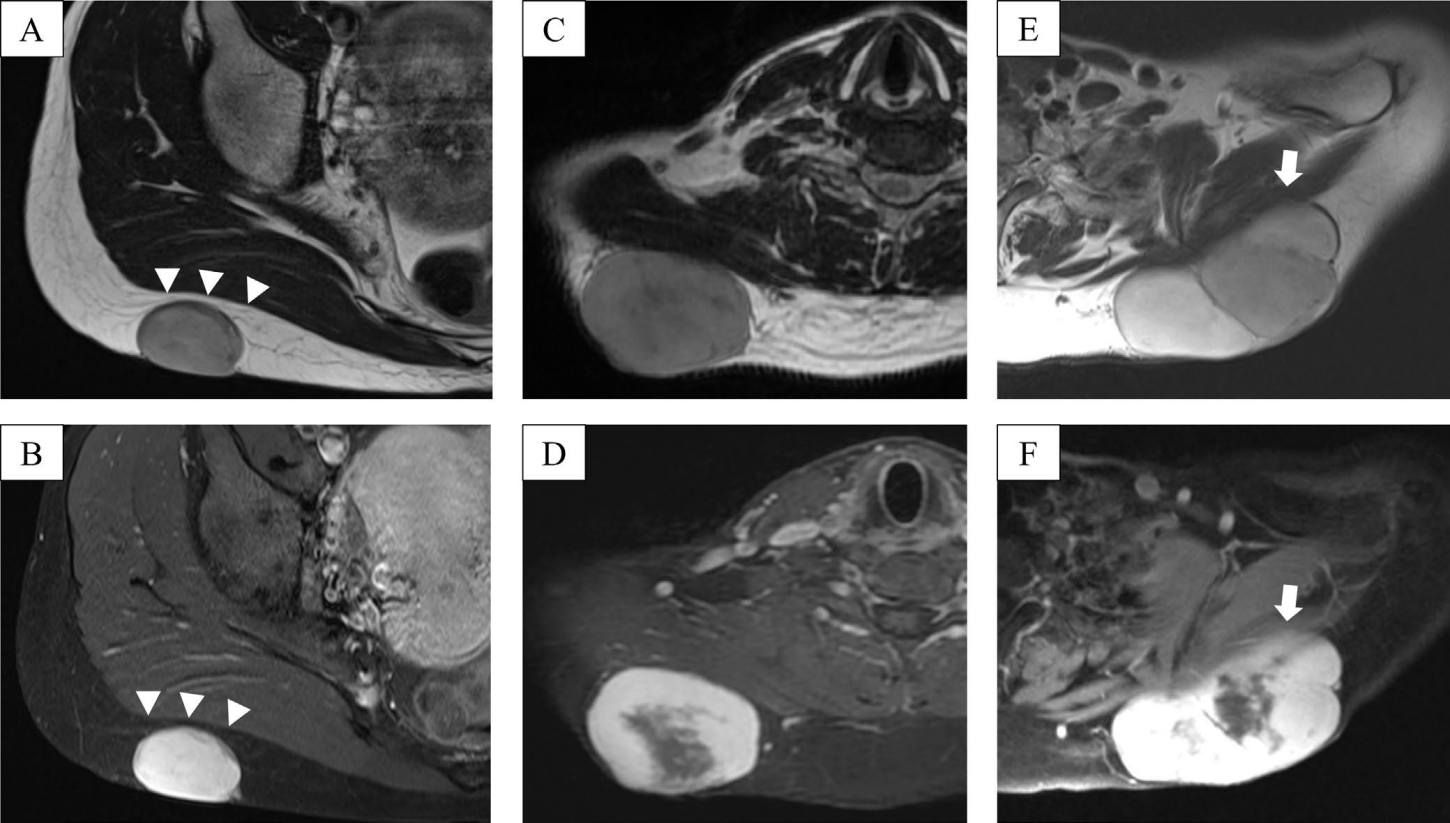
Assessment of the relationship between the tumor and the fascia on preoperative MRI. (1) Not contacting fascia (gluteal lesion). On T2‐weighted imaging (A) and contrast‐enhanced fat‐suppressed T1‐weighted imaging (B), interposed fat tissue is observed between the tumor and the muscle (arrowheads). (2) Contacting fascia (back lesion). T2‐weighted imaging (C) and contrast‐enhanced fat‐suppressed T1‐weighted imaging (D) show no interposed fat tissue between the tumor and the muscle. (3) Beyond fascia (back lesion). On T2‐weighted imaging (E) and contrast‐enhanced fat‐suppressed T1‐weighted imaging (F), continuous signal change extending into the muscle is observed, suggesting intramuscular invasion (arrow).

For deep assessment, the relationship between the tumor and fascia was classified into three categories: no contact with fascia, contact with fascia, and beyond fascia (Figure 2). When a clear fat layer was present between the tumor and muscle, the lesion was classified as “no contact.” Loss of the fat plane with tumor abutting the fascia was classified as “contact.” Continuous tumor signal extending into muscle was classified as “beyond fascia.” In head lesions, where clear fascial structures are absent, the periosteum was used as an anatomical surrogate for the fascia.

In all cases, resection including the fascia was performed regardless of imaging findings. Therefore, no cases with fascia preservation were included in this study.

### 2.3 Pathological Evaluation

**FIGURE 3:**
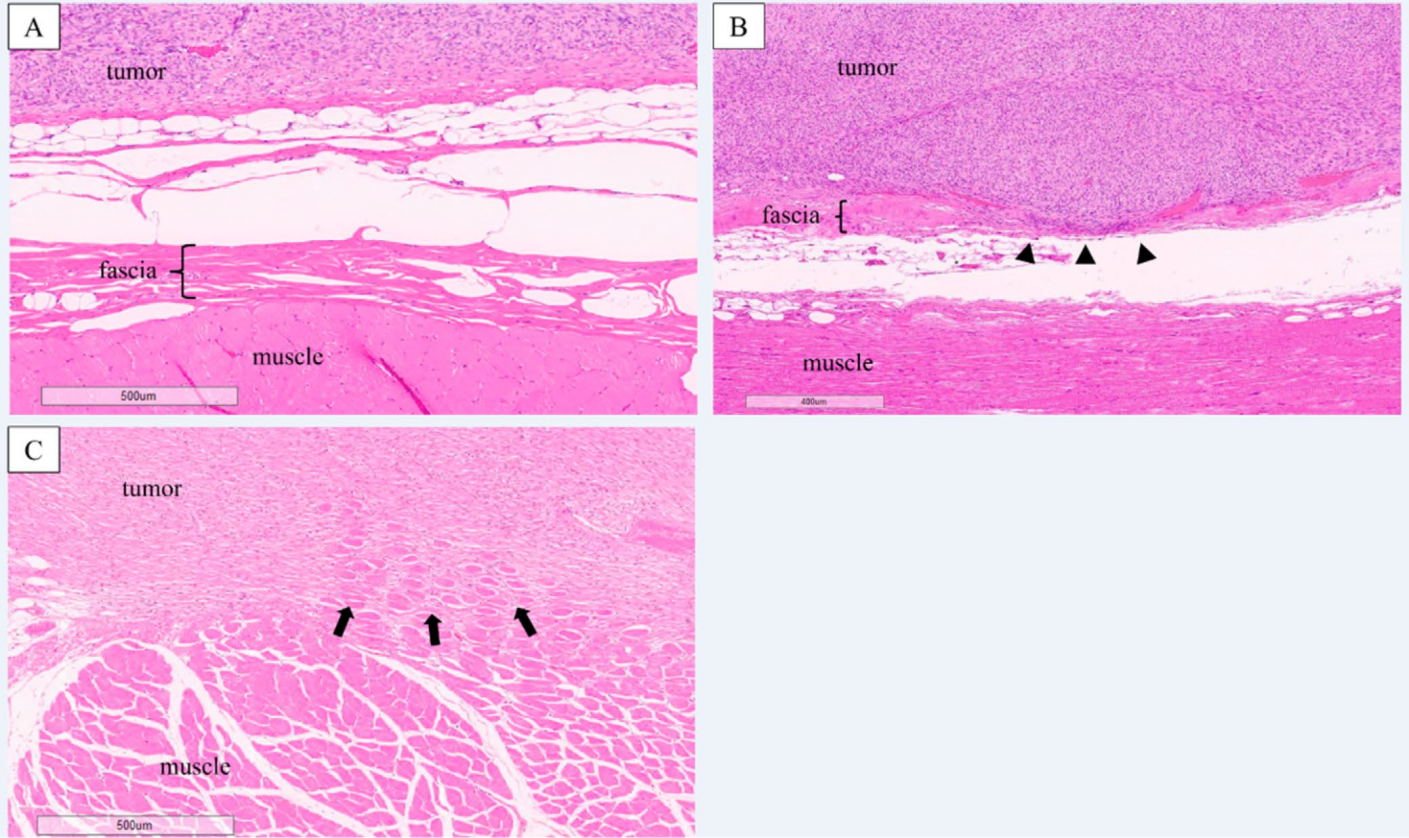
Patterns of pathological deep invasion. (A) Confined to the subcutaneous tissue. Interposed subcutaneous fat tissue is present between the tumor and the fascia. (B) Fascia invasion. The tumor invades into the fascia (arrowhead). (C) Muscle invasion. The tumor invades into the muscle, with disruption of the fascial structure (arrow).

Pathological evaluation was performed on resected specimens. The lateral margin was defined as the shortest distance from the tumor to the surgical resection edge. Deep invasion was classified into three categories based on the deepest extent of tumor infiltration: confined to subcutaneous tissue, fascia invasion, and muscle invasion (Figure 3).

### 2.4 Statistical Analysis

Continuous variables are presented as median and range or interquartile range (IQR). In both lateral and deep margin analyses, cases without pathological residual tumor were excluded because assessment of tumor extent was not feasible. For lateral margin analysis, the difference between pathological margin and planned margin (pathological − planned margin) was calculated. Paired comparisons were performed using the Wilcoxon signed‐rank test, and correlation was assessed using Spearman's rank correlation coefficient. Comparisons between two independent groups were performed using the Mann–Whitney *U* test, and comparisons among three groups were performed using the Kruskal–Wallis test. Categorical variables were compared using Fisher's exact test. For deep margin analysis, pathological deep invasion was categorized into two groups: confined to subcutaneous tissue and invasion into fascia or muscle.

The association between preoperative imaging findings and pathological deep invasion was evaluated using Fisher's exact test, and odds ratios (OR) with 95% confidence intervals (CI) were calculated.

A two‐sided *p* value < 0.05 was considered statistically significant. Statistical analyses were performed using R (version 4.5.2).

## 3 Results

### 3.1 Patient Characteristics

A total of 42 patients were analyzed. Patient characteristics stratified by primary treatment and unplanned excision are summarized in Table 1. The median age was 46 years (range, 4–92 years), and 25 patients (60%) were male. Histological diagnoses included 35 cases (83%) of conventional DFSP and seven cases (17%) of FS‐DFSP. Tumor location was most commonly the trunk (25 cases, 60%), followed by extremities (12 cases, 29%) and head and neck (5 cases, 12%). Among head and neck cases, two were located in the scalp.

**TABLE 1:** Patient characteristics according to clinical presentation.

Variable	Primary treatment (*n* = 21)	After unplanned excision (*n* = 21)	*p*
Age at first visit, median (range), years	46.7 (4.0–92.0)	45.3 (21.3–78.5)	0.821
Sex			
Male	16	9	0.058
Female	5	12	
Histological subtype			
DFSP	17	18	1.000
FS‐DFSP	4	3	
Tumor location			
Trunk	16	9	0.076
Extremities	3	9	
Head and neck	2	3	
Tumor size^a^, median (range), cm	5.1 (2.6–9.3)	2.5 (0.8–7.4)	< 0.001
Follow‐up period, median (range), years	4.3 (1.0–12.1)	6.4 (1.2–15.2)	0.119
Preoperative imaging before previous excision			
Available	—	5	
Not available	—	16	

Abbreviations: DFSP; dermatofibrosarcoma protuberans; FS‐DFSP; fibrosarcomatous dermatofibrosarcoma protuberans.

^a^ Tumor size was evaluated only in cases with assessable lesions on preoperative imaging.

At initial presentation, 21 patients (50%) underwent primary treatment at our institution, whereas 21 patients (50%) were referred after unplanned excision at another institution. Among referred cases, preoperative imaging before previous excision had been performed in only five cases (24%). The median tumor size was significantly larger in the primary treatment group than in the unplanned excision group (5.1 cm vs. 2.5 cm, *p* < 0.001). No statistically significant differences were observed between the two groups regarding age, sex, histological subtype, tumor location, or follow‐up period.

### 3.2 Lateral Margin Evaluation

**FIGURE 4:**
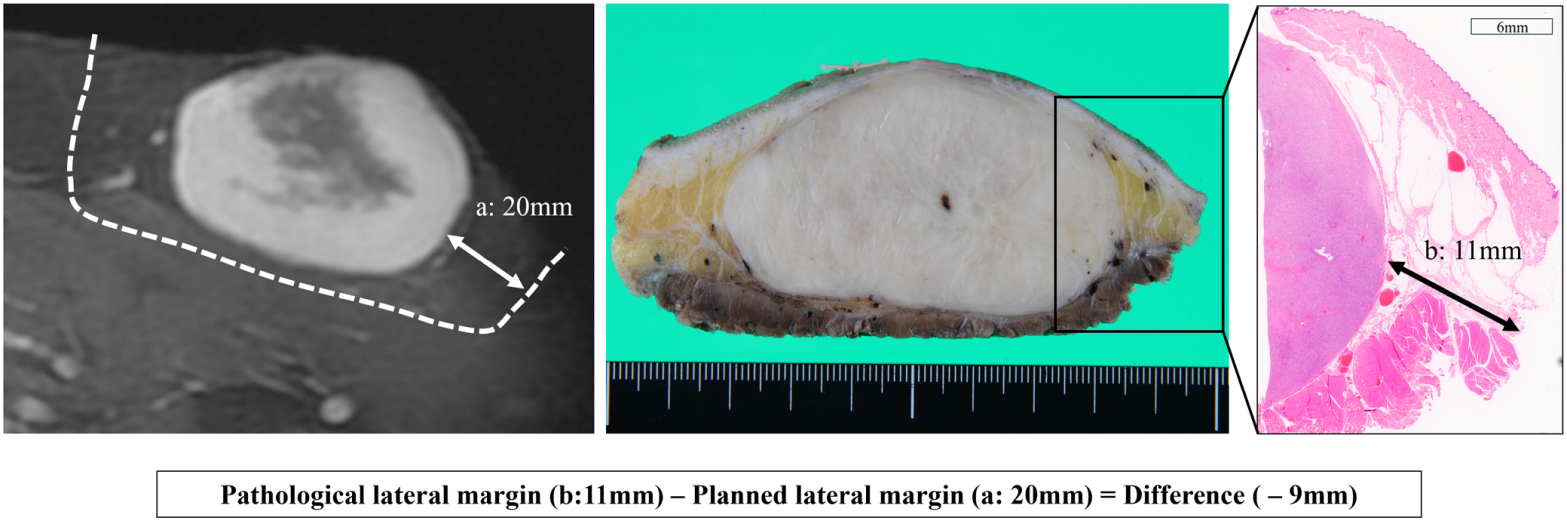
Method for calculating the difference between the pathological lateral margin and the preoperative planned lateral margin.

**FIGURE 5:**
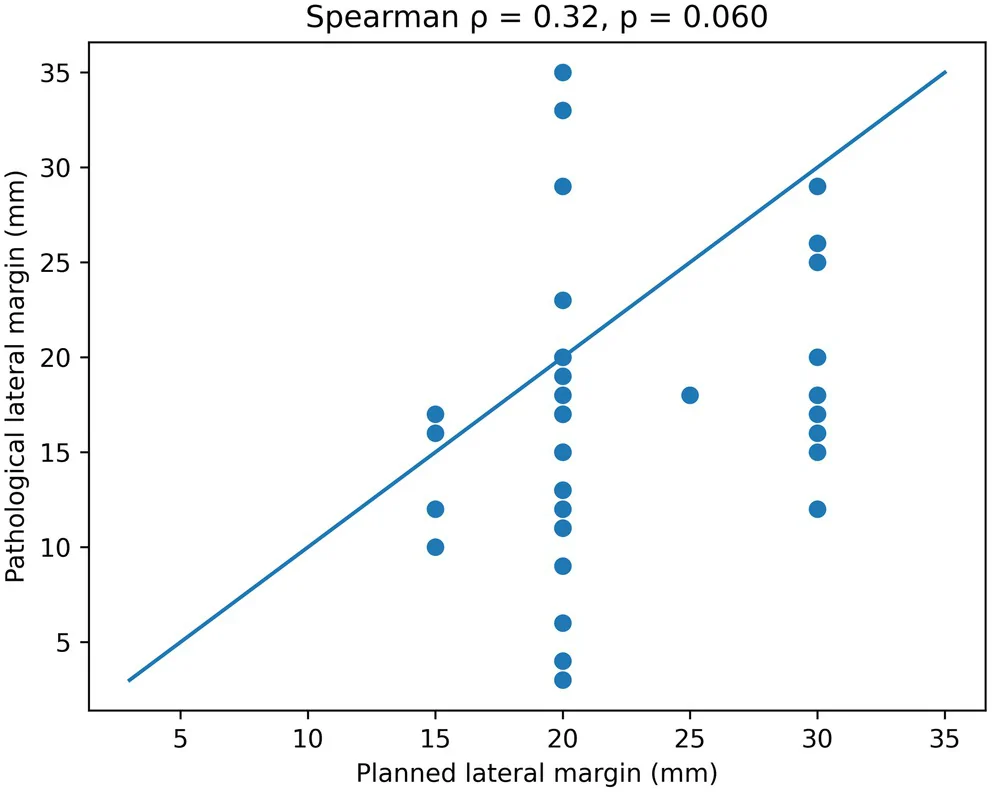
Correlation between the preoperative planned lateral margin and the pathological lateral margin. Each point represents one case. The diagonal line indicates the line of identity (*y* = *x*). A weak positive correlation was observed (*ρ* = 0.32), but it did not reach statistical significance (*p* = 0.060).

Preoperative MRI sequences included contrast‐enhanced T1‐weighted fat‐suppressed images in 33 cases and STIR sequences in nine cases (Table 2). The median planned lateral margin was 20 mm (range, 15–30 mm). Lateral margin analysis was performed in 36 patients after excluding cases in which no tumor was identified pathologically. The median pathological lateral margin was 16.5 mm (range, 3–35 mm). The median difference (pathological − planned margin) was −5 mm (IQR −10.25 to −1 mm), with a range of −18 to +15 mm (Figure 4). Wilcoxon signed‐rank test showed that planned margins were significantly larger than pathological margins (*p* < 0.001). A weak positive correlation was observed between planned and pathological margins (*ρ* = 0.32), but it did not reach statistical significance (*p* = 0.060) (Figure 5). When analyzed separately, correlation was weak in the primary group (*ρ* = 0.22, *p* = 0.33) and moderate in the unplanned excision group (*ρ* = 0.45, *p* = 0.09). There was no significant difference in margin discrepancy between the two groups (*p* = 0.33). In subgroup analysis according to tumor location, the median margin discrepancy was −5 mm in the trunk, −5 mm in the extremities, and −11 mm in the head and neck. No statistically significant difference was observed among locations (*p* = 0.552). In the comparison based on contrast‐enhanced MRI, the margin discrepancy tended to be smaller in the contrast‐enhanced group compared with the non‐contrast group (median −5 mm vs. 7 mm), although the difference was not statistically significant (*p* = 0.279).

**TABLE 2:** Planned and pathological lateral surgical margins and subgroup analyses.

Variable	Value	*p*
Planned lateral margin, median (range), mm	20 (15–30)	
Pathological lateral margin, median (range), mm	16.5 (3–35)	
Difference (pathological margin − planned margin), median (IQR, range), mm	−5 (IQR −10.25 to −1, range −18 to +15)	< 0.001^a^

Subgroup analysis of margin discrepancy		
Subgroup	Median difference (range), mm	*p*
Clinical status at first visit		
Primary treatment	−6 (−18 to +10)	0.33^b^
After unplanned excision	−5 (−17 to +15)	
Tumor location		
Trunk	−5 (−17 to +3)	0.552^c^
Extremities	−5 (−13 to +15)	
Head and neck	−11 (−18 to +9)	

*Note:* Difference was calculated as pathological margin minus planned margin. Negative values indicate pathological margins smaller than planned margins, whereas positive values indicate pathological margins larger than planned margins.

^a^ Wilcoxon signed‐rank test.

^b^ Mann–Whitney *U* test.

^c^ Kruskal–Wallis test.

### 3.3 Deep Margin Evaluation

Preoperative imaging classified tumors as no contact with fascia in 19 cases, contact in 19 cases, and beyond fascia in four cases (Table 3). All patients underwent resection including the fascia. The actual depth of resection included fascia in 22 cases and fascia with muscle in 20 cases. Pathological evaluation showed 26 cases confined to subcutaneous tissue, five cases with fascia invasion, and five cases with muscle invasion. Six cases had no residual tumor and were excluded from deep margin analysis.

**TABLE 3:** Deep margin evaluation: Preoperative imaging, surgical resection, and pathological findings.

Preoperative imaging evaluation	Surgical resection	Pathological finding				Total
		Subcutaneous only	Fascia invasion	Muscle invasion	No residual tumor	
Not contacting fascia	Fascia	12	0	0	3	15
	Fascia + muscle	4	0	0	0	4
	Subtotal	16	0	0	3	19
Contacting fascia	Fascia	2	3	0	2	7
	Fascia + muscle	8	2	2	0	12
	Subtotal	10	5	2	2	19
Beyond fascia/suspected deep invasion	Fascia + muscle	0	0	3	1	4
	Subtotal	0	0	3	1	4
Total		26	5	5	6	42

Among the 36 evaluable cases, no deep invasion was observed in the fascia noncontact group. In contrast, deep invasion was observed in cases classified as fascia‐contacting or beyond. Fisher's exact test showed a significant association (*p* < 0.001). The odds of deep invasion were significantly higher in the fascia‐contacting/beyond group (OR 27.0, 95% CI 1.5–483).

### 3.4 Additional Resection and Oncologic Outcomes

Among the 42 patients, 21 (50%) underwent additional wide resection after unplanned excision performed at another institution, and residual tumor was identified in 15 of these cases (71%). Among these referred cases, most had undergone previous excision without imaging‐based surgical planning.

All 42 patients ultimately achieved R0 resection. Reconstructive procedures were required in selected cases following wide local excision, particularly in anatomically sensitive regions. No local recurrence was observed during follow‐up. Distant metastases occurred in two patients (5%), both of whom had FS‐DFSP. Oncologic outcomes were as follows: continuous disease‐free (CDF) in 37 patients, no evidence of disease (NED) in four patients, and death of other disease (DOOD) in one patient.

## 4 Discussion

In this study, we retrospectively evaluated the relationship between preoperative imaging‐based surgical margin planning and pathological tumor invasion in patients with DFSP treated with wide local excision (WLE). A weak positive correlation was observed between planned lateral margins based on imaging and pathological margins, although it did not reach statistical significance. This finding suggests that preoperative imaging does not precisely predict tumor extent but may still provide clinically useful information for surgical planning.

In the surgical treatment of DFSP, the most important factor for local control is not the absolute width of the margin, but whether pathological complete resection (R0) is achieved. Previous studies by Heuvel et al. [3] and Chen and Jiang [13] have demonstrated that local recurrence and outcomes are associated with the adequacy of resection margins. Cohort studies by Alshaygy et al. [1] and Huis In 't Veld et al. [4] also support the importance of negative margins. In the present study, R0 resection was achieved in all cases, and no local recurrence was observed during follow‐up, which is consistent with these previous reports.

The optimal lateral margin in WLE has long been debated, and margins of 20–50 mm have traditionally been recommended [6]. In this study, the median planned lateral margin was 20 mm, and the median pathological margin was 16.5 mm. The median discrepancy was relatively small (−5 mm), and the range was within −18 to +15 mm. Although statistical correlation between planned and pathological margins was limited, the magnitude of discrepancy remained within a clinically acceptable range. In addition, part of this discrepancy may be attributable to tissue shrinkage during formalin fixation and pathological processing, although this effect was not directly evaluated in the present study.

Importantly, the maximum underestimation was 18 mm, indicating that the discrepancy did not exceed 20 mm in any case. This suggests that a lateral margin of approximately 20 mm provides a reasonable safety margin to account for variability in tumor infiltration. In addition, a weak positive correlation between planned and pathological margins suggests that imaging‐based planning may reflect tumor extent to a certain degree.

On the other hand, some cases showed larger pathological margins than planned margins, suggesting possible overestimation of tumor extent based on imaging. Given the infiltrative nature of DFSP, setting margins on the safe side is reasonable; however, these findings indicate that staged margin‐controlled approaches may be considered in selected cases.

Comparison based on contrast‐enhanced MRI showed a tendency toward smaller margin discrepancies in contrast‐enhanced cases; however, no statistically significant difference was observed. This may be influenced by the limited number of non‐contrast cases. Nevertheless, in all cases, the discrepancy was within a clinically acceptable range, suggesting that a lateral margin of approximately 20 mm based on preoperative imaging is practical in clinical settings regardless of imaging conditions.

With regard to deep surgical margins, no standardized guidelines specific to DFSP have been established. In this study, we clearly distinguished between preoperative imaging‐based assessment of deep invasion, actual surgical resection depth, and pathological findings.

Cases evaluated as fascia‐contacting or beyond on preoperative imaging showed a higher tendency for deep invasion compared with fascia noncontact cases (OR = 27.0, 95% CI 1.5–483). However, the wide confidence interval indicates statistical uncertainty due to the limited number of cases, and this finding should be interpreted with caution. Therefore, although definitive conclusions cannot be drawn, these results suggest that preoperative imaging may be useful for stratifying the risk of deep invasion.

Furthermore, stratification based on imaging may also provide useful information for determining surgical strategy. In this study, no deep invasion was observed in cases classified as fascia noncontact, whereas invasion into fascia or muscle was observed in fascia‐contacting or beyond cases. These findings suggest that, in cases without fascia contact, resection including the fascia may be sufficient for local control, whereas in cases with fascia contact or suspected muscle invasion, deeper resection may be considered from a safety perspective.

However, all cases in this study underwent resection including the fascia, and the safety of fascia preservation or optimization of muscle resection extent was not directly evaluated. This should be considered when interpreting the results.

The deep margin evaluation strategy used in this study is consistent with surgical principles that emphasize anatomical barriers such as fascia and periosteum [15]. In head lesions, where fascial structures are not clearly defined, the periosteum was used as an alternative boundary. The periosteum can also function as a physical barrier to tumor invasion, and this approach is considered theoretically consistent.

This study included both conventional DFSP and FS‐DFSP. FS‐DFSP is known to have higher malignant potential and a higher risk of local recurrence and distant metastasis compared with conventional DFSP [12, 16]. However, the pattern of local infiltration is similar between the two, and the surgical strategy of a 20‐mm lateral margin and fascia‐based deep resection may be applicable to both.

In this study, all cases achieved R0 resection and no local recurrence was observed, suggesting that this surgical strategy is appropriate for local control even in FS‐DFSP. However, distant metastases occurred in FS‐DFSP cases, indicating differences in biological behavior despite adequate local control.

Therefore, while the surgical approach may be similar, careful postoperative surveillance is required for FS‐DFSP.

DFSP is characterized by infiltrative growth even when clinically well‐demarcated. Inadequate initial excision and unplanned excision are known to increase the risk of residual tumor and positive margins [1, 2, 4]. In addition, insufficient margins are strongly associated with local recurrence [3]. In the present study, residual tumor was identified in 71% of patients who underwent additional resection after unplanned excision, highlighting the importance of appropriate initial surgical planning. Notably, most referred cases had undergone previous excision without preoperative imaging‐based planning. This may partly explain the high residual tumor rate observed in the present study and further supports the importance of appropriate preoperative imaging assessment in DFSP. Although half of the cases in this study involved unplanned excision, subgroup analysis showed no significant difference in margin discrepancy between primary and unplanned excision cases, suggesting that the validity of the analysis was not substantially affected.

Mohs surgery and PDEMA are theoretically superior techniques but require specialized intraoperative assessment systems and are not widely available. Slow Mohs surgery, which applies the concept of margin‐controlled resection using paraffin sections, may be an alternative approach without requiring intraoperative frozen section analysis [17, 18]. Particularly in head and neck cases, where functional preservation is important, this approach may help avoid excessive resection [19]. In the present subgroup analysis, the margin discrepancy in head and neck cases was not significantly different from that in other locations; however, the number of head and neck cases was limited, and the maximum underestimation was 18 mm. Therefore, the present findings do not support routine reduction of the lateral margin below approximately 20 mm in head and neck lesions. In this study, the absence of deep invasion in fascia noncontact cases and the possibility of overestimation of lateral margins suggest that slow Mohs surgery may be considered in selected cases. However, slow Mohs surgery also has limitations, including longer treatment duration and increased patient burden, and should not be applied universally. Appropriate patient selection based on tumor size, location, imaging findings, and functional considerations is essential.

This study has several limitations. First, it is a retrospective single‐institution study with a limited sample size. Second, all cases underwent fascia resection, and the safety of fascia preservation was not evaluated. Third, the number of cases with deep invasion was limited, resulting in wide confidence intervals and limited statistical robustness. In addition, although no local recurrence was observed, the follow‐up period in some cases was relatively short. Therefore, the present findings should not be interpreted as indicating that a 20‐mm lateral margin completely eliminates the risk of recurrence.

Future multicenter studies are required to further validate optimal margin strategies.

## 5 Conclusion

Preoperative imaging provides clinically useful information for surgical margin planning in DFSP. A lateral margin of approximately 20 mm appears to be sufficient to achieve R0 resection, and fascia‐based evaluation may support individualized deep resection strategies.

## Funding

The authors have nothing to report.

## Ethics Statement

This study design was approved by the institutional ethics review board of Shinshu University School of Medicine (approval number: 6763). This study did not involve animal experiments. The requirement for informed consent was waived due to the retrospective nature of the study, and an opt‐out approach was adopted in accordance with institutional review board approval.

## Conflicts of Interest

The authors declare no conflicts of interest.

## Data Availability

The data that support the findings of this study are available on request from the corresponding author. The data are not publicly available due to privacy or ethical restrictions.
